# Dopamine D1–D2 Receptor Heteromer in Dual Phenotype GABA/Glutamate-Coexpressing Striatal Medium Spiny Neurons: Regulation of BDNF, GAD67 and VGLUT1/2

**DOI:** 10.1371/journal.pone.0033348

**Published:** 2012-03-12

**Authors:** Melissa L. Perreault, Theresa Fan, Mohammed Alijaniaram, Brian F. O'Dowd, Susan R. George

**Affiliations:** 1 Centre for Addiction and Mental Health, Toronto, Ontario, Canada; 2 Department of Pharmacology and Toxicology, University of Toronto, Toronto, Ontario, Canada; 3 Department of Medicine, University of Toronto, Toronto, Ontario, Canada; Institute for Interdisciplinary Neuroscience, France

## Abstract

In basal ganglia a significant subset of GABAergic medium spiny neurons (MSNs) coexpress D1 and D2 receptors (D1R and D2R) along with the neuropeptides dynorphin (DYN) and enkephalin (ENK). These coexpressing neurons have been recently shown to have a region-specific distribution throughout the mesolimbic and basal ganglia circuits. While the functional relevance of these MSNs remains relatively unexplored, they have been shown to exhibit the unique property of expressing the dopamine D1–D2 receptor heteromer, a novel receptor complex with distinct pharmacology and cell signaling properties. Here we showed that MSNs coexpressing the D1R and D2R also exhibited a dual GABA/glutamate phenotype. Activation of the D1R–D2R heteromer in these neurons resulted in the simultaneous, but differential regulation of proteins involved in GABA and glutamate production or vesicular uptake in the nucleus accumbens (NAc), ventral tegmental area (VTA), caudate putamen and substantia nigra (SN). Additionally, activation of the D1R–D2R heteromer in NAc shell, but not NAc core, differentially altered protein expression in VTA and SN, regions rich in dopamine cell bodies. The identification of a MSN with dual inhibitory and excitatory intrinsic functions provides new insights into the neuroanatomy of the basal ganglia and demonstrates a novel source of glutamate in this circuit. Furthermore, the demonstration of a dopamine receptor complex with the potential to differentially regulate the expression of proteins directly involved in GABAergic inhibitory or glutamatergic excitatory activation in VTA and SN may potentially provide new insights into the regulation of dopamine neuron activity. This could have broad implications in understanding how dysregulation of neurotransmission within basal ganglia contributes to dopamine neuronal dysfunction.

## Introduction

In contrast to classical thinking which depicts the dopamine D1 and D2 receptors (D1R and D2R) as being completely segregated to dynorphin (DYN)-expressing and enkephalin (ENK)-expressing striatonigral and striatopallidal pathways respectively, a growing accumulation of functional [1]–[6], and neuroanatomical [7]–[14] evidence now indicates that a physiologically relevant subset of medium spiny neurons (MSNs) exhibits a mixed phenotype, coexpressing the dopamine D1R and D2R in addition to substance P (SP)/DYN and ENK. Indeed, it has recently been reported that these MSNs exhibit a region-specific distribution throughout the mesolimbic and basal ganglia circuits [13]. More specifically, while a relatively low number of D1R-containing MSNs express the D2R (∼6%) in caudate putamen (CP), higher coexpression levels are evident in ventral pallidum and entopeduncular nucleus, with the highest levels in the nucleus accumbens shell (NAc) (∼17–34%) and globus pallidus (∼60%) [13], [15]. In addition, as D1R and D2R coexpression has been reported to occur selectively at presynaptic, but not postsynaptic terminals [13], together these findings suggest that MSNs coexpressing the D1R and D2R may have a unique physiological function at a local level as well as distal effects through their efferent projections that potentially impact on both the striatonigral and striatopallidal pathways.

Although the physiological relevance of D1R and D2R coexpressing MSNs remains relatively unexplored, these neurons have also been shown to have the unique property of expressing the dopamine D1R–D2R heteromer, a novel receptor complex with discrete pharmacology and cell signaling properties [12], [14], [16], [17]. Specifically, the D1R–D2R heteromer has been shown to be distinct from its constituent receptors in that it is coupled to Gq/11 to activate phospholipase C and generate intracellular calcium release, representing a novel signaling pathway directly linking dopamine action to calcium [12], [17]. More recently, the activity of the D1R–D2R heteromer has been shown to be upregulated in rat striatum following repeated amphetamine administration and in the globus pallidus of patients who had schizophrenia [13], signifying a potential role for this receptor complex in pathophysiologies involving elevated dopamine transmission. In this study we sought to further elucidate the importance of the dopamine D1R–D2R heteromer in mediating neurotransmission within regions of the basal ganglia and associated mesolimbic system by assessing the expression of proteins known to be involved in GABA or glutamate production and release. We showed that MSNs coexpressing D1R and D2R exhibited a unique dual GABA/glutamate phenotype and activation of the D1R–D2R heteromer by the selective agonist SKF 83959 in these neurons differentially and simultaneously regulated the expression of proteins involved in GABA and glutamate activity in regions of the mesolimbic and nigrostriatal pathways.

## Results

### D1R and D2R coexpressing MSNs also express both GABA and glutamate

Striatal MSNs, which make up approximately 95% of all neurons in this region, are consistently characterized as being solely GABAergic. However we found that the subtype of GABA MSNs that coexpressed the D1R and D2R also exhibited a glutamatergic phenotype. We assessed coexpression of the D1R and D2R with protein markers for GABA and glutamate neuron identification, glutamate decarboxylase 67 (GAD67) and the vesicular glutamate transporters 1 and 2 (VGLUT1, VGLUT2). The specificity of the D1R and D2R antibodies has been strictly validated and previously reported [13]. Specifically, dopamine receptor antibodies for the D1R and D2R were tested using the five dopamine receptors (D1–D5) expressed individually in HEK293 cells, and testing was also performed in striatal tissue of D1R or D2R gene-deleted mice where we showed no reactivity of the D1R or D2R antibody respectively. When the primary D1R and D2R antibodies and the relevant secondary antibodies were combined, no cross-excitation of the secondary fluorophores was evident and controls were also performed in the absence of the primary or secondary antibodies to exclude cross-reactivity.

**Figure 1:**
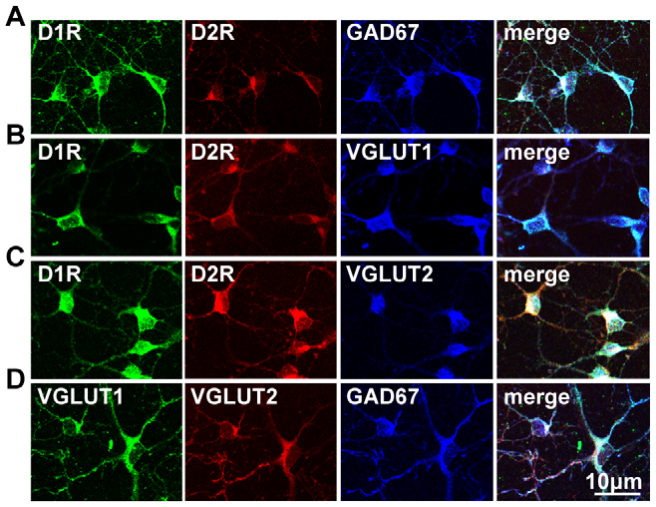
Dopamine D1R and D2R colocalized in dual phenotype GABA/glutamate-expressing MSNs in cultured neonatal striatal neurons. Confocal images revealed D1R and D2R colocalization with the GABA neuronal marker, GAD67 (top row), and the glutamate markers, VGLUT1 and VGLUT2 in striatal neurons cultured 7–10 days. Scale bar 10 µm.

**Figure 2:**
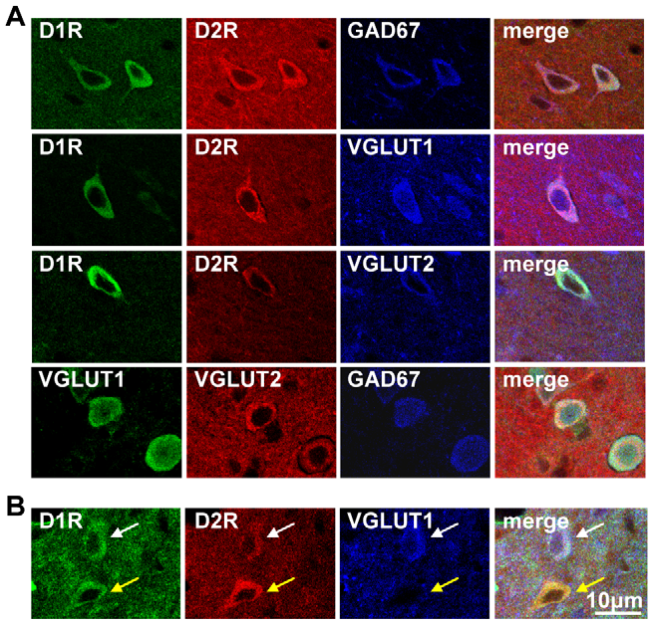
Dopamine D1R and D2R colocalized in GABA/glutamate-coexpressing MSNs in adult rat NAc. (**A**) Confocal images revealed D1R and D2R colocalization with GAD67 (top row) and VGLUT1 and VGLUT2 in NAc core. GAD67 was also shown to colocalize with VGLUT1 and VGLUT2 in these neurons (bottom row). (**B**) Colocalization of the D1R and D2R with VGLUT1 (white arrows) and in the absence of VGLUT1 (yellow arrows) in NAc shell. Scale bar 10 µm.

**Figure 3:**
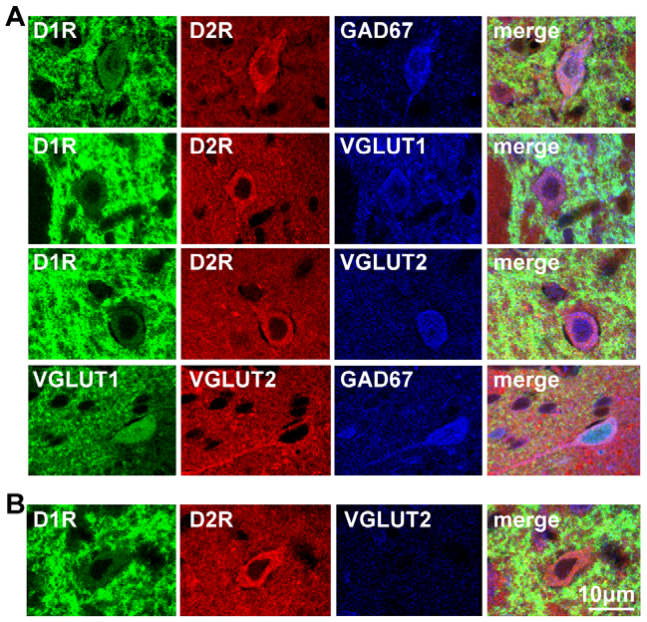
Dopamine D1R and D2R colocalized in GABA/glutamate-coexpressing MSNs in adult rat CP. (**A**) Confocal images showing D1R and D2R colocalization with GAD67 (top row), and VGLUT1 and VGLUT2 in CP. GAD67 also colocalized with VGLUT1 and VGLUT2 in CP (bottom row). Note the high levels of dendritic staining for the D1R in this region. (**B**) A neuron showing colocalization of the D1R and D2R in with in the absence of VGLUT2. Scale bar 10 µm.

It was observed in cultured neonatal striatal neurons, almost all of which exhibit the D1R/D2R-DYN/ENK phenotype [10], [14], that these neurons also coexpressed GAD67, as well as VGLUT1 and VGLUT2 (Fig. 1). To determine whether this mixed GABA/glutamate phenotype was retained in D1R/D2R-DYN/ENK neurons into adulthood, D1R and D2R coexpression with GAD67, VGLUT1 and VGLUT2 was examined in adult rat NAc (Fig. 2) and CP (Fig. 3). We showed in these regions that almost all neurons coexpressing the D1R and D2R also expressed GAD67, VGLUT1 or VGLUT2 (Figs. 2A and 3A), signifying that these MSNs were unique in potentially having both inhibitory and excitatory capabilities in adult striatum as well as in neonatal striatal neurons. A very small minority of neurons that coexpressed the D1R and D2R in the absence of VGLUT1 (Fig. 2B) or VGLUT2 (Fig. 3B) was visualized, suggesting that these neurons either did not express VGLUT1 or VGLUT2 or, alternatively, may have expressed the VGLUT subtype not examined in that particular experiment. Nonetheless, these results suggest that the large majority of D1R/D2R coexpressing neurons expressed both VGLUT1 and VGLUT2. Given the prevalence of these D1R/D2R-DYN/ENK coexpressing MSNs throughout both the striatopallidal and striatonigral pathways of the basal ganglia [13], these findings emphasize the potential importance of these mixed phenotype neurons not only in the regulation of thalamic output, but additionally in the regulation of its associated neuronal connections to regions rich in dopamine cell bodies such as to the ventral tegmental area (VTA) and substantia nigra (SN).

### Brain region-specific modification of GABA and glutamate production and/or vesicular uptake by dopamine D1R–D2R heteromer

**Figure 4:**
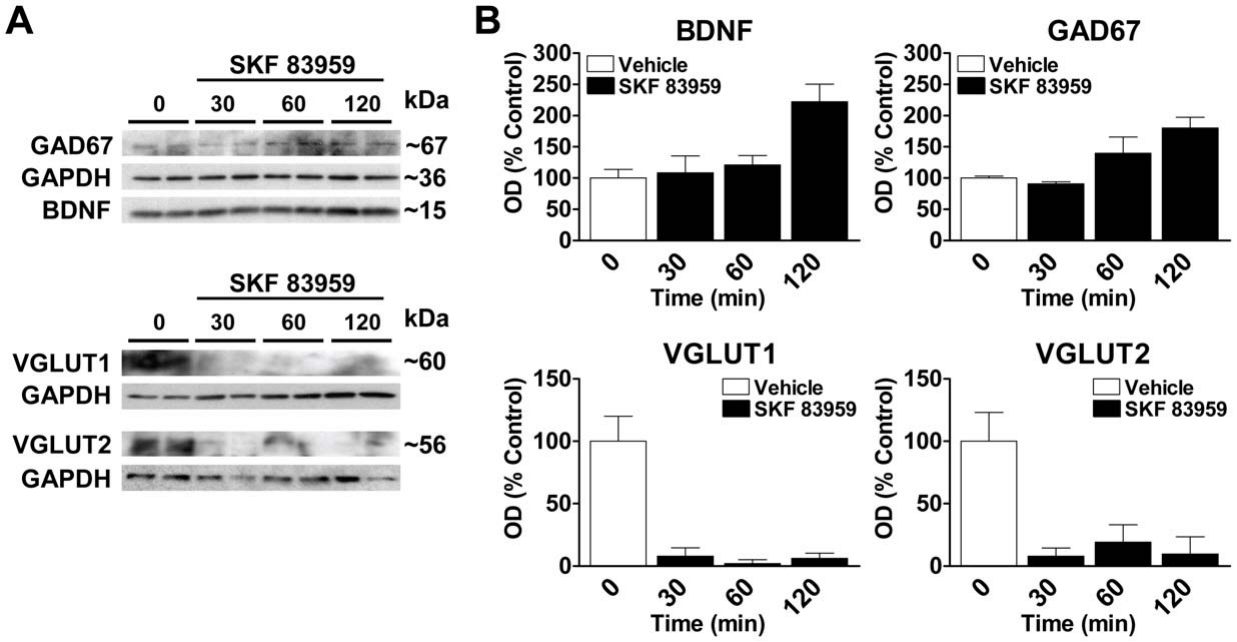
Enhanced BDNF and GAD67, and reduced VGLUT1/2 expression following D1R–D2R heteromer activation. (**A**) Representative blots depicting the effects treatment of striatal neuronal cultures with vehicle or the D1R–D2R heteromer-selective agonist SKF 83959 (100 nM) for 30, 60 or 120 min on BDNF, GAD67, VGLUT1 and VGLUT2 expression. (**B**) Treatment of the neuronal cultures with SKF 83959 for 120 min increased the expression of BDNF and GAD67. In contrast, the expression of VGLUT1 and VGLUT2 was reduced with a 30 min treatment of SKF 83959. Values shown are mean ± S.D.

**Figure 5:**
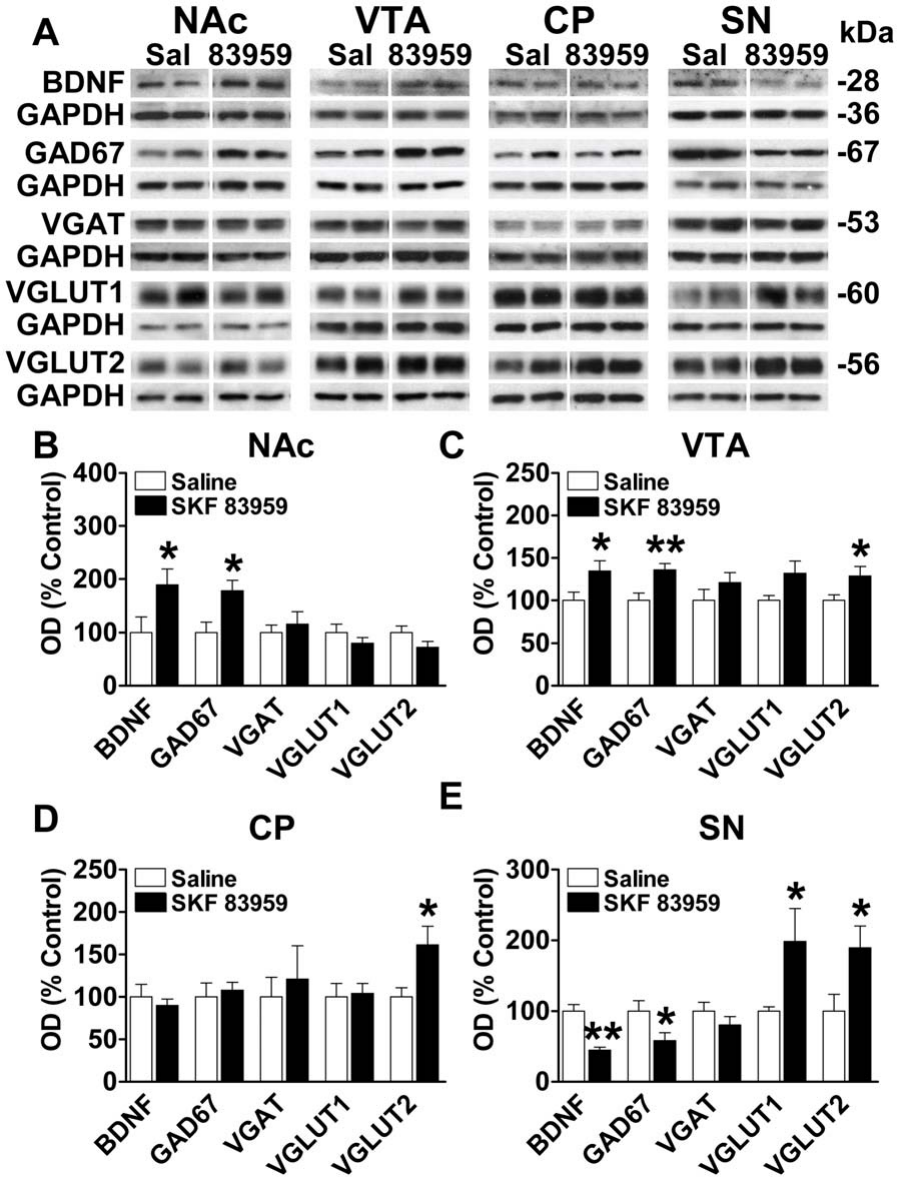
D1R–D2R heteromer discretely regulates the expression of proteins involved in GABA or glutamate activity. (**A**) Representative blots depicting the effects of a single injection of the D1R–D2R heteromer agonist SKF 83959 (1.5 mg/kg, sc) on BDNF, GAD67, VGAT, VGLUT1 and VGLUT2 expression in NAc, CP, VTA and SN (n = 8–9 rats/group). GAPDH was used as a loading control. (**B, C**) SKF 83959 increased expression of BDNF and GAD67 in NAc and VTA. No drug effects were observed on VGAT, VGLUT1 or VGLUT2 levels in NAc, while a significant increase in VGLUT2 only was seen in VTA. (**D, E**) SKF 83959 had no effect on BDNF and GAD67 expression in CP, but diminished expression in SN. Increased levels of VGLUT2 were also evident in response to SKF 83959 in CP, with both VGLUT1 and VGLUT2 being elevated in SN. Bars shown represent means ± s.e.m. and are expressed as a percentage of saline controls. *P<0.05, ** *P*<0.01.

Neurons that coexpress the D1R and D2R have a unique function in that they express the dopamine D1R–D2R heteromer, a novel receptor complex linked to Gq-mediated intracellular calcium release and brain-derived neurotrophic factor (BDNF) production [12], [14], [16], [17]. We have shown that the D1R–D2R heteromer is localized to both cell soma and at presynaptic terminals in NAc and CP [13] a finding suggestive of a possible role in presynaptic GABA or glutamate neurotransmission from these neurons, as well as the potential for both local and distal effects on other neuronal subtypes such as those individually expressing the D1R or D2R. To further elucidate the impact of D1R–D2R heteromer activity on overall changes in GABA and glutamate activity, we assessed the effects of the selective heteromer agonist SKF 83959 on the expression of GAD67, VGLUT1 and VGLUT2 in cultured neonatal striatal neurons. These proteins are highly specific neuronal markers and additionally provide a suitable index of GABA and glutamate neurotransmission given their role in neurotransmitter production or presynaptic vesicular uptake. We first examined GAD67, the major enzyme involved in neuronal GABA production and the expression of which has been shown to be associated with BDNF signaling [18], [19]. Treatment of cultured striatal neurons with 100 nM SKF 83959 led to a time-dependent increase in BDNF and GAD67 expression as well as a decline in the expression of VGLUT1 and VGLUT2 (Fig. 4). To determine the effects of D1R–D2R heteromer activation on these proteins *in vivo*, we next administered an acute systemic injection of SKF 83959 and examined the expression of BDNF, GAD67, VGLUT1 and VGLUT2, as well as the vesicular GABA transporter (VGAT), in regions of the mesolimbic system and basal ganglia of the brain (Fig. 5). Systemic activation of the D1R–D2R heteromer by SKF 83959 led to increased BDNF and GAD67 expression in the NAc and VTA {NAc: BDNF *P* = 0.016, GAD67 *P* = 0.021; VTA: BDNF *P* = 0.044, GAD67 *P* = 0.006} (Fig. 5B and 5C). In contrast, in SN a significant decrease in the expression of both proteins was observed {BDNF, *P* = 0.0001; GAD67 *P* = 0.041} with no changes in CP (Fig. 5D,E). SKF 83959 did not alter the expression of VGAT in any of the regions examined. We showed no effect of SKF 83959 on VGLUT1 or VGLUT2 expression in NAc (Fig. 5B) and a modest but significant increase of VGLUT2 in VTA {*P* = 0.050} (Fig. 5C). However, an elevation in the expression of both VGLUT1 and VGLUT2 in SN {VGLUT1 *P* = 0.042; VGLUT2 *P* = 0.012} (Fig. 5E), and VGLUT2 in CP {*P* = 0.025} was observed (Fig. 5D). A direct relationship between vesicular glutamate uptake by VGLUTs and glutamate release has been demonstrated [20], [21]. These results are indicative of a potential role for the D1R–D2R heteromer in mediating glutamate release in regions of the nigrostriatal pathway with no effect on GABA. In contrast, the D1R–D2R heteromer had a direct role in GABA production in regions of the mesolimbic pathway with little effect on glutamate.

### Region-specific regulation of pCaMKII and pERK by the D1R–D2R heteromer

**Figure 6:**
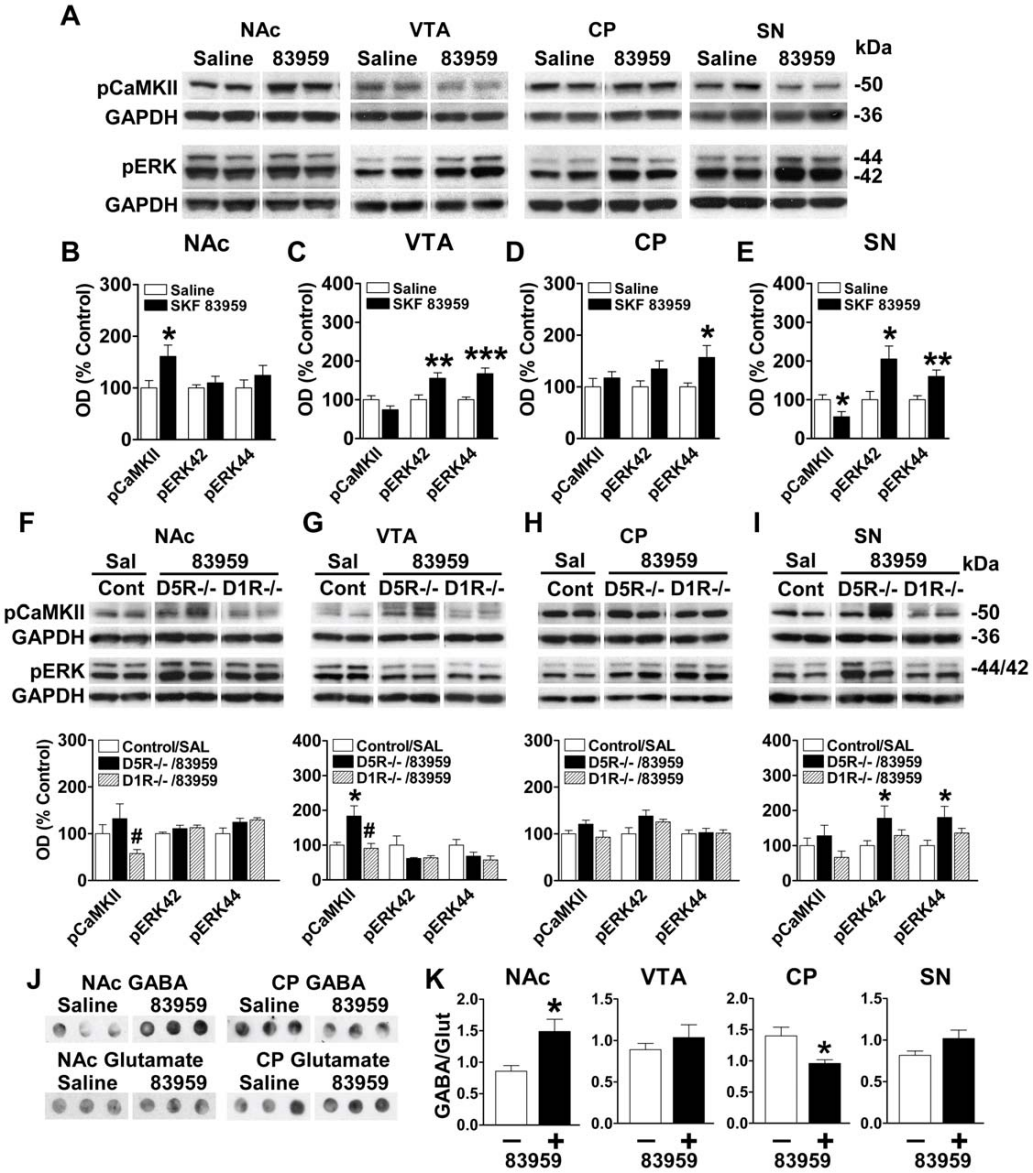
Region-specific regulation of pCaMKII, pERK, GABA and glutamate expression by the dopamine D1R–D2R heteromer. (**A**) Representative blots depicting the effects of a single systemic injection of SKF 83959 (1.5 mg/kg, sc) on pCaMKII and pERK expression in NAc, CP, VTA and SN (n = 8 rats/group). GAPDH was used as a loading control. (**B–E**) SKF 83959 increased CaMKII phosphorylation in NAc, had no effects in VTA and CP, and reduced pCaMKII levels in SN. Phosphorylation of ERK42 and ERK44 was elevated in both VTA and SN, and pERK44 levels were increased in CP. Data are expressed as a percentage of saline controls. (**F–I**) In the presence of SKF 83959 D1R−/− mice exhibited significantly lower levels of pCaMKII than D5R−/− mice in NAc and VTA. There were no changes in pERK42 or pERK44 expression by SKF 83959 in either gene-deleted strain in these regions. SKF 83959 did not alter pCaMKII expression in either the D1R−/− or D5R−/− mice in CP or SN. However, SKF 83959 induced a significant increase in SN pERK expression in D5R−/− mice, but not D1R−/− mice. (**J**) Representative dot blots showing effects of SKF 83959 on total GABA and glutamate levels in NAc (left panels) and CP (right panels). (**K**) SKF 83959 increased GABA levels, relative to glutamate, in NAc, but reduced levels in CP. No drug effects were observed in VTA or SN (N = 8–9 rats/group). Bars shown represent means ± s.e.m. *P<0.05, ** *P*<0.01, *** *P*<0.001, #*P*<0.05 compared to D5R−/− mice.

The expression of BDNF has been shown to be regulated by calcium/calmodulin kinase IIα (CaMKII) activation via phosphorylation at Thr^286^ [22], while phosphorylation of extracellular regulated kinase (ERK) has been implicated in VGLUT expression [23]. We showed a significant increase in pCaMKII expression in NAc following acute systemic SKF 83959 administration {*P* = 0.042} (Fig. 6A,B). It should be noted, that while SKF 83959 activates the D1R–D2R heteromer, the drug also activates the D5 receptor (D5R). Although D5R expression in NAc and CP is relatively low, being localized predominantly to cholinergic interneurons [24] that comprise only ∼1–2% of neurons in striatum, we did confirm that the increased NAc pCaMKII levels were induced by the D1R–D2R heteromer, and not by D5R activation, as the expression of pCaMKII was not elevated in response to SKF 83959 in mice gene deleted for the D1R (D1R−/− mice) (Fig. 6F). An increase in pCaMKII in VTA by SKF 83959 was not evident (Fig. 6C) despite the increased BDNF expression in this region (Fig. 5C). We postulate that this lack of an overall region-wide effect of SKF 83959 on VTA pCaMKII expression may have been the result of a D5R-mediated suppression of pCaMKII levels as, in the absence of the D5R (in D5R−/− mice), there was a significant increase in pCaMKII in response to the drug {*P* = 0.036} (Fig. 6G). The idea of an attenuating effect of the D5R on pCaMKII is also consistent with the results found in SN, a region with relatively higher abundance of D5R [25], and which showed an overall reduction in pCaMKII expression following SKF 83959 (Fig. 6E). In VTA, CP, and SN, regions that showed increased expression of VGLUT1 and/or VGLUT2 (Fig. 5), SKF 83959 induced an increase in the levels of pERK42 and pERK44 {VTA: pERK42/44 *P* = 0.010/0.001; CP: pERK44 *P* = 0.047; SN: pERK42/44 *P* = 0.018/0.007} (Fig. 6C–E), a finding that supports the previously documented role for pERK in VGLUT expression [23]. Both the D1R–D2R heteromer and D5R appeared to be involved in pERK expression as the induction of pERK by SKF 83959 was absent in both D1R−/− and D5R−/− mice in VTA and CP (Fig. 6G,H). In SN, however (Fig. 6I), the D1R–D2R heteromer was solely responsible for the increased expression of pERK as these effects were absent in the D1R−/− mice, but present in mice gene-deleted for the D5R {pERK42/44 *P* = 0.050/0.045}.

### Total striatal GABA and glutamate levels altered following D1R–D2R heteromer activation

Thus far, these findings indicated that activation of the dopamine D1R–D2R heteromer by SKF 83959 resulted in the pathway-specific regulation of protein expression associated with GABA and glutamate activity, with predominantly increased expression of proteins involved with GABA activation in regions of the mesolimbic pathway along with an associated elevation in proteins involved with glutamate activity in regions of the nigrostriatal pathway. To further characterize this, we assessed total levels of GABA and glutamate expression in NAc, VTA, CP and SN (Fig. 6J,K) following activation of the D1R–D2R heteromer. SKF 83959 induced a significant increase in the expression of GABA, relative to glutamate, in NAc {*P* = 0.019} signifying a potential shift towards GABA neurotransmission in this region. In contrast, a significant reduction in the ratio of GABA to glutamate expression was evident in CP {*P* = 0.029}, indicative of a shift toward glutamate transmission. No changes in the relative expression of GABA were present in SN or VTA following SKF 83959.

### D1R–D2R heteromers in NAc shell discretely alter the expression of proteins involved in GABA and glutamate activity in SN and VTA

**Figure 7:**
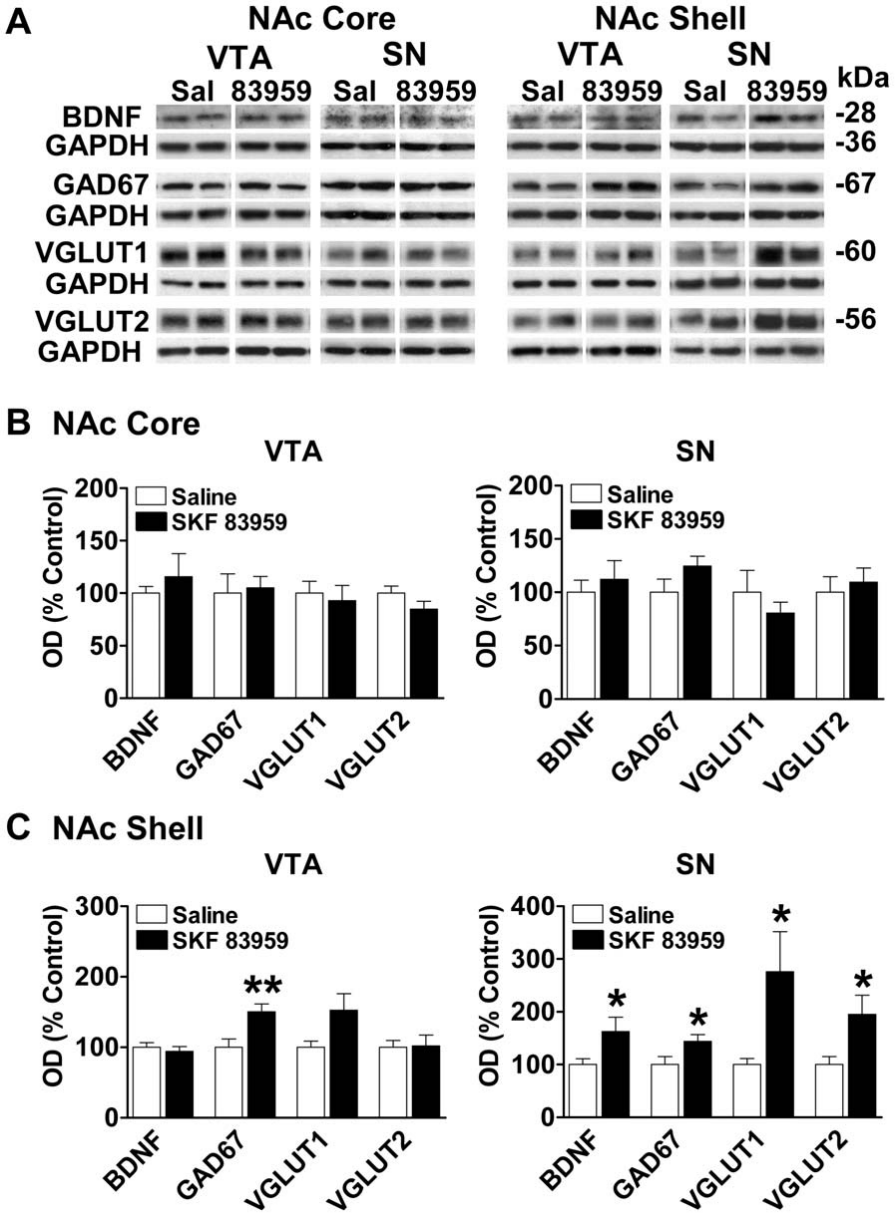
NAc core versus shell activation of dopamine D1R–D2R heteromer differentially regulates protein expression in VTA and SN. (**A**) Representative blots depicting the effects of a single intra-NAc core or shell injection of SKF 83959 (0.75 µg/0.5 µl unilateral) on BDNF, GAD67, VGLUT1 and VGLUT2 expression in VTA and SN (n = 9 rats/group). GAPDH was used as a loading control. (**B**) There was no effect of an intra-NAc core injection of SKF 83959 on protein expression in VTA (left panel) or SN (right panel). (**C**) SKF 83959 administration into NAc shell induced a significant increase in GAD67 expression in VTA, but had no effect on BDNF or VGLUT expression (left panel). In contrast, SN showed elevated levels of BDNF, GAD67, VGLUT1 and VGLUT2 (right panel). Bars shown represent means ± s.e.m. and are expressed as a percentage of controls. *P<0.05, ** *P*<0.01.

**Figure 8:**
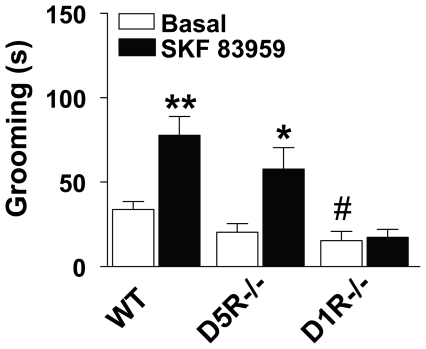
Grooming induced by SKF 83959 is mediated by the dopamine D1R–D2R heteromer. A single systemic injection of SKF 83959 (1.5 mg/kg, sc) induced a significant elevation in the amount of time spent grooming (n = 7 mice/group). This effect was also present in mice gene-deleted for the D5R (D5R−/−) but absent in mice gene-deleted for the D1R (D1R−/−). D1R−/− mice, but not D5R−/− mice, also exhibited reduced basal levels of grooming compared to wildtype (WT). (Strain {F(2,36) = 12.1, P<0.001}; Drug {F(1,36} = 17.8, P<0.001}; Strain x Drug {F(2,36) = 4.0, P<0.03}). Bars shown represent means ± s.e.m. and are expressed in seconds (s). *P<0.05, ** *P*<0.01 compared to basal levels within the same strain. #*P* = 0.025 compared to basal wildtype.

As the NAc shows a relatively high abundance of D1R–D2R heteromers [13], we next sought to determine the importance of D1R–D2R heteromers localized to NAc core and shell in regulating GABA- and glutamate-related protein expression in VTA and SN (Fig. 7). Systemic administration of SKF 83959 has been shown previously to induce orofacial movements and grooming in rats [13], [26]. It was noted that SKF 83959 injection directly into NAc shell, but not NAc core, resulted in the development of orofacial movements such as facial twitching and teeth grinding in anaesthetized animals. Upon awakening, these animals additionally exhibited elevated grooming behaviour, an effect mediated by the D1R–D2R heteromer as grooming behaviour was absent in D1R−/− mice {*P* = 0.81}, but retained in D5R −/− mice {*P* = 0.019} (Fig. 8). Activation of the D1R–D2R heteromer by SKF 83959 in NAc core had no effect on expression of BDNF, GAD67, VGLUT1 or VGLUT2 in the VTA or SN (Fig. 7A,B). Activation of the D1R–D2R heteromer in NAc shell however, induced significant increases in GAD67 in VTA {*P* = 0.009} (Fig. 7C, *left panel*) and both BDNF and GAD67 in SN {BDNF, *P* = 0.039; GAD67 *P* = 0.044} (Fig. 7C, *right panel*). These effects in SN were opposite to that observed with the systemic injection of SKF 83959, supporting a negative role for SN D5R in the pCaMKII-BDNF-GAD67 signaling cascade. In addition, a NAc shell injection also elevated the expression of both VGLUT1 {*P* = 0.036} and VGLUT2 {*P* = 0.035} supporting that the systemic effects of SKF 83959 on VGLUT and pERK expression in SN were mediated by the D1R–D2R heteromer. We cannot presently determine whether there is a specific contribution of D1R–D2R heteromer-induced GABA- versus glutamate-related protein expression in the subregions of SN, namely the SN pars compacta (SNc) and SN pars reticulata (SNr). Nonetheless, as activation of the D1R–D2R heteromer in NAc shell appears to mediate both inhibitory and excitatory outputs to SN, but only inhibitory outputs to VTA, these findings indicate that the regulation of dopamine neurons in these regions by the dual GABA/glutamate MSNs is fundamentally different.

## Discussion

In the present study we have identified a striatal MSN subtype coexpressing GABA and glutamate as well as the D1R and D2R together with DYN and ENK, and likely having both inhibitory and excitatory capabilities. In cultured neonatal striatal neurons the majority of neurons exhibited this mixed D1R/D2R-GABA/glutamate expressing phenotype, and a significant fraction of these coexpressing neurons was retained into adulthood in NAc. It was further shown in adult rat that the activity of these D1R/D2R-GABA/glutamate MSNs was differentially regulated by the dopamine D1R–D2R heteromer, and that this regulation occurred in a pathway-specific manner. These findings not only demonstrate the existence of a novel source of glutamate in the basal ganglia circuitry, but additionally provide new insights into the neuroanatomy and physiology of basal ganglia functioning and thereby having the potential to improve the understanding of the plasticity underlying neuronal communication.

### Mixed phenotype D1R/D2R-DYN/ENK-GABA/glutamate neurons in basal ganglia

It has been previously documented that the D1R and D2R are coexpressed exclusively in a subset of striatal neurons that coexpress the neuropeptides DYN and ENK [13]. In the present study it was shown that these coexpressing MSNs are also positive for markers of GABA and glutamate, indicative of neurons with dual inhibitory and excitatory regulatory properties, and a finding supported by a previous neuroanatomical study that showed some striatal projection neurons possessed a high affinity uptake system for glutamate and aspartate [27]. The pervasive presence of these D1R/D2R-DYN/ENK-GABA/glutamate coexpressing neurons in neonatal striatum suggests that these neurons may contribute to the ontogeny of neuronal development, which has been previously suggested for developing hippocampal GABA/glutamate-expressing granular neurons [28]. However, we additionally demonstrated that a proportion of these MSNs retained this mixed phenotype into adulthood, a finding suggestive of a role for these specialized MSNs in mediating neurotransmission in adult brain. Indeed, we showed that these neurons were physiologically active in adult striatum and provided a substrate for the direct coupling of dopamine signaling to GABA and glutamate production and release. Specifically, D1R/D2R-coexpressing MSNs differentially regulated the expression of proteins involved in GABA and glutamate activation in the NAc and VTA, as well as the CP and SN, effects that were mediated by the dopamine D1R–D2R heteromer and that are indicative of a significant role for these neurons in the control of striatal MSN signaling. Furthermore, as we have recently reported that D1R and D2R coexpressing neurons exhibit a region-dependent distribution within both the striatonigral and striatopallidal pathways of the basal ganglia circuitry [13], we propose that there exists a subcircuitry of D1R/D2R-DYN/ENK-GABA/glutamate MSNs, which interconnects the basal ganglia nuclei, and which has the potential to impact on mesolimbic and thalamic output. Further studies will be required to clarify the specific neuronal subtypes that contribute to the alterations in protein expression reported herein, neurons that may have included MSNs expressing individual D1R or D2R, cholinergic neurons, and cortical glutamatergic afferents.

### Regulation of mixed phenotype MSNs by the D1R–D2R heteromer in regions of the mesolimbic and nigrostriatal pathways

We identified a novel GABA/glutamate coexpressing efferent projection from NAc shell, regulated by the dopamine D1R–D2R heteromer, which mediated GABA-related protein expression in VTA. Similarly, systemic activation of the dopamine D1R–D2R heteromer by SKF 83959 induced CaMKII activation and BDNF and GAD67 expression in the NAc and VTA, a finding also indicative of increased GABAergic tone in regions of the mesolimbic pathway of the brain. As the activity of VTA dopamine neurons is modulated by plasticity related to inhibitory and excitatory inputs, and dysregulation of mesolimbic dopamine signaling has been widely shown to be pivotal to neuropsychiatric dopamine disorders, the present results may be indicative of a mechanistic link between dopamine D1R–D2R heteromer-induced signaling and disorders involving abnormal dopamine transmission.

The idea of a role for the D1R–D2R heteromer in contributing to disorders characterized by abnormal dopamine signaling is supported by studies showing an integral involvement of CaMKII, BDNF or GAD67 in the pathological processes underlying drug addiction and schizophrenia. For instance, NAc shell CaMKII has been previously shown to be a critical component underlying cocaine seeking by serving as a biochemical link between dopamine and glutamate [29]. In addition, NAc and VTA BDNF signaling mediates the magnitude of the reward responses to cocaine [30]–[32], an interesting finding given the present results, and previous reports, linking the dopamine D1R–D2R heteromer to calcium signaling, CaMKII activation and BDNF expression in NAc [12], [14], [17]. Along the same lines, the pathogenesis of schizophrenia has been repeatedly associated with GABA dysfunction in a number of regions including striatum, an effect mediated in part by reduced expression of GAD67 [33], [34], and which we postulate may contribute to the increased VTA dopamine neuronal activation inherent in the disorder. Interestingly, mice deficient in CaMKII, a protein that may potentially contribute to gene expression of GAD67 via phosphorylation of the transcription repressor protein MeCP2 [22], [35], [36], also displayed attributes similar to animal models of schizophrenia [37]. As we have also previously shown an increase in the activation state of the D1R–D2R heteromer in rat striatum following repeated amphetamine administration, or in the globus pallidus of patients who had schizophrenia [13], together these findings suggest that further research into a role for the D1R–D2R heteromer in these disorders involving dopamine dysfunction is warranted.

Following intra-NAc shell activation of the D1R–D2R heteromer there was increased expression of BDNF and GAD67 in SN, that was also concurrent with stimulation of VGLUT1 and VGLUT2 expression. At first glance, this dual increase in both GABA- and glutamate-related protein expression may appear to be redundant. However it is unlikely these changes simply negate one another as alterations in neurotransmission would most likely occur in distinct localized areas, such as at discrete neuronal synapses or within different SN subregions. Indeed, neuroanatomical studies examining striatonigral projection neurons coexpressing D1R/D2R or SP/ENK have shown that these striatonigral efferents terminate in both subregions of SN, namely the SNr and SNc [11], [38]. We therefore suggest that D1R–D2R heteromer-induced changes in GABA and glutamate activity in SN may possibly occur via discrete projections from striatal efferents to SNr and SNc.

Although the relative involvement of the SNr and SNc in mediating the observed effects on D1R–D2R heteromer-induced protein expression in SN could not be elucidated in the present study, the SNr has been shown to be a pivotal region in the stimulation of jaw movements and grooming in rodents [39], [40], behaviors that manifested following systemic [13] or intra-NAc shell SKF 83959 administration as shown herein. Interestingly, jaw movements elicited by ventral striatal costimulation of the D1R and D2R are regulated by SNr NMDA receptor activation [39], a finding thus linking increased D1R–D2R heteromer-induced SNr glutamate, and VGLUT1 and VGLUT2 expression, to this behavior in rats. In addition, in SNc, studies have shown neuroprotective effects of the D1R–D2R heteromer agonist SKF 83959 on dopamine neurons following MPTP lesioning in mice [41]. We postulate that these protective effects of SKF 83959 on SNc neurons may be reflective of increased D1R–D2R heteromer-induced BDNF expression, as protective effects of BDNF on SNc dopamine neurons in parkinsonian animals have been reported [42]–[44]. Notably, SKF 83959 has been associated with strong anti-parkinsonian effects, with an absence of dyskinesias, in non-human primate and rodent animal models of Parkinson's disease [45]–[48], and significantly reduced L-Dopa-induced dyskinesias in 6-OHDA lesioned rats [48]. Therefore we posit that the D1R–D2R heteromer may mediate, at least in part, the therapeutic effects of L-dopa, but not the deleterious side effects induced by this treatment, implicating this receptor complex as a valid novel therapeutic target in Parkinson's disease.

In summary, we demonstrated that in basal ganglia there exists a subset of striatal MSNs that exhibit a novel mixed D1R/D2R-DYN/ENK-GABA/glutamate phenotype, which may have projections, direct or indirect, from the NAc shell to areas rich in dopamine cell bodies where the mesolimbic and nigrostriatal pathways originate. We further showed that these neurons were regulated by the dopamine D1R–D2R heteromer, a receptor complex that exhibited selective, but differential control of the expression of proteins associated with GABA and glutamate activity in the NAc and VTA as well as the CP and SN. That the dopamine D1R–D2R heteromer had the ability to differentially regulate D1R/D2R-DYN/ENK/-GABA/glutamate MSNs, neurons with postulated intrinsic inhibitory and excitatory functions, in discrete regions of the basal ganglia emphasizes a potential role of this receptor complex in mediating the plasticity underlying the transition between GABAergic and glutamatergic dominance. This could potentially have broad implications in furthering the understanding of the pathophysiology and therapeutic management of mesolimbic and basal ganglia disorders, such as schizophrenia, drug addiction and Parkinson's disease.

## Materials and Methods

### Neuronal Cultures

Neonatal rat striata (1 day of age) were trypsinized in Hanks' balanced salt solution (HBSS) with 0.25% trypsin and 0.05% DNase (Sigma) at 37°C, and cells were washed three times in HBSS with 12 mM MgSO4. Cells were dissociated in DMEM with 2 mM glutamine and 10% FBS and plated at 2×10^5^ cells per poly-L-lysine-coated well (Sigma; 50 µg/mL). The next day, media were changed to Neurobasal medium with 50× B27 Supplement and 2 mM glutamine (Invitrogen). On day 3 of culture, 5 µM cytosine arabinoside was added to inhibit glial cell proliferation. Half of the medium was changed every 3 days.

### Animals

Sixty adult male Sprague-Dawley rats (Charles River, Canada) and forty adult gene-deleted (D1R−/− or D5R−/−) or control mice were used. D1R−/− and D5R−/− mice were congenic, having been backcrossed 12 times (N12). All procedures involving animals complied with the guidelines described in the Guide to the Care and Use of Experimental Animals (Canadian Council on Animal Care, 1993), and were approved by the Animal Care Ethics Committee of the University of Toronto (permit numbers 20008894 and 20008895).

### Drugs

SKF 83959 hydrobromide (Tocris Bioscience) was dissolved in 0.9% saline containing 5% DMSO. Systemic injections were given subcutaneously at a dose of 1.5 mg/kg. For non-drug injections, an equivalent volume of saline was administered. All injections were administered at a volume of 1.0 ml/kg for rats and 5.0 ml/kg for mice. For intra-NAc core or shell injections SKF 83959 was dissolved in DMSO and aCSF at a volume of 0.75 µg/0.5 µl.

### Fluorescence Immunohistochemistry

Fluorescence immunohistochemistry was performed as previously described [14]. Paraformaldehyde-fixed striatal neurons or coronal sections from untreated rat brain CP and NAc were incubated with primary antibodies (1∶200) for 60 hours at 4°C (D1R, Sigma-Aldrich; D2R, Chemicon; GAD67, VGLUT1, VGLUT2, Millipore). Specificity of the dopamine receptor antibodies for the D1R and D2R have been previously tested and were validated in D1R or D2R gene-deleted mice [13]. To minimize background and prevent cross-excitation of the secondary antibody-linked fluorophores, only three primary antibodies were used on the cultured neurons or tissue at any given time. Images were obtained using an Olympus Fluoview 1000 confocal microscope at 63× magnification.

### Grooming

Animals were administered SKF 83959 (1.5 mg/kg, sc) and placed immediately inside an empty cage similar in dimensions to the home cage. Grooming activity was then monitored for 30 minutes. The measurement of grooming behavior followed a previously described protocol [49] with the following modifications. The animal's grooming was scored randomly for 30 seconds for a total of 4 minutes (2 minutes sampled from the first 15 minutes of testing and 2 minutes sampled from the last 15 minutes of testing).

### Immunoblot

Fifteen or ninety minutes following SKF 83959 administration brains were rapidly removed and tissue from the NAc, CP, SN and VTA dissected and flash frozen until ready for use. Tissue was suspended in cell lysis buffer and 10–30 micrograms of protein were incubated in sample buffer for 3 minutes at 95°C. Samples were separated by SDS-PAGE on a 10% tris-glycine gel and electroblotted on PVDF transfer membrane for 2.5 hours. For GABA and glutamate, a specific volume of homogenate, standardized to total protein content, was pipetted directly onto nitrocellulose membrane and BSA was used as a negative control. Membranes were blocked and incubated overnight at 4°C with gentle shaking with primary antibody to BDNF 1∶10000, GAD67 1∶6000, VGLUT1 1∶10000, and VGLUT2 1∶10000 (Millipore), to VGAT 1∶5000, pCaMKII 1∶5000, (Pierce), to pERK1(42) and pERK2(44) 1∶3000, GABA 1∶4000, glutamate 1∶10000 (Sigma Aldrich). Membranes were then washed in TBS-Tween and incubated for 2 hours at room temperature with species-specific secondary antibody (Bio-Rad Laboratories, Hercules, CA, USA). Antibody labeling of proteins were detected with enhanced chemiluminescence (Mandel Scientific or Millipore) and signal intensity was quantified using Zeiss AxioVision4 software.

### Surgery

Rats were anesthetized using isoflurane, administered an injection of the analgesic ketoprofen (5 mg/kg, sc) and secured in a stereotaxic frame. Each rat was unilaterally injected into NAc core or shell with SKF 83959 (0.75 µg/0.5 µl) according to the following stereotaxic anterior–posterior (AP), mediolateral (ML), and dorsoventral (DV) coordinates: NAc Core: AP +1.8 mm, ML ±1.6 mm, DV −7.0 mm; NAc Shell: AP +1.8 mm, ML ±0.8 mm, DV −7.5 mm. AP and ML coordinates were taken from bregma, DV coordinates from skull surface (Paxinos and Watson, 1998). Following removal from the stereotaxic frame, animals were monitored for involuntary orofacial movements, and upon recovery for grooming behaviour. Ninety minutes following injection animals were decapitated, brains rapidly removed, and injection sites into Nac core or shell confirmed. SN and VTA were then dissected and flash frozen for use in immunoblot analysis.

### Data Analysis

All values are reported as mean ± s.e.m. Comparisons of means for protein expression was performed by the Student's *t* test (two-tailed, unpaired). For the grooming data, the statistical significance of each dependent measure was first evaluated using an ANOVA with Strain and Drug as the between subjects factors followed by post-hoc Student's *t* tests. The immunoblot data was collected by densitometry and the main dependent variable was Grey x area of band, expressed as a percent of saline controls. Computations were performed using the SPSS/PC+ statistical package.
